# [Retracted] Interleukin-17 promotes the development of cisplatin resistance in colorectal cancer

**DOI:** 10.3892/ol.2026.15611

**Published:** 2026-04-22

**Authors:** Guolong Sui, Yingna Qiu, Haijuan Yu, Qingbin Kong, Baowen Zhen

Oncol Lett 17: 944–950, 2019; DOI: 10.3892/ol.2018.9645

Following the publication of the above paper, it was drawn to the Editor's attention by a concerned reader that certain of the western blot data shown in Fig. 5C on p. 949 had already appeared previously in a pair of articles written by different authors at different research institutes, which were published in the journals *Oncology Letters* and *Molecular Medicine Reports.*

Owing to the fact that the contentious data in the above article had already been published prior to its submission to *Oncology Letters*, the Editor has decided that this paper should be retracted from the Journal. The authors were asked for an explanation to account for these concerns, but the Editorial Office did not receive a reply. The Editor apologizes to the readership for any inconvenience caused.

